# Focused Solar-Induced Construction of Activated Solar Carbon@Carbon Fiber Coaxial Electrode from Waste Carbon Fiber-Reinforced Polymer and Its Supercapacitor Performance

**DOI:** 10.3390/molecules30153093

**Published:** 2025-07-24

**Authors:** Chongjun Zhao, Tenghui Huang, Yingying Rong, Yanyu Guo, Puqi Geng, Chunhua Zhao

**Affiliations:** Key Laboratory for Ultrafine Materials of Ministry of Education, Shanghai Key Laboratory of Advanced Polymeric Materials, School of Materials Science and Engineering, East China University of Science and Technology, Shanghai 200237, China; y30220891@mail.ecust.edu.cn (T.H.); ryy991011@163.com (Y.R.); ecustguany@163.com (Y.G.); y82220315@mail.ecust.edu.cn (P.G.)

**Keywords:** focused sunlight, photothermal effect, reclaimed carbon fiber, C@CF coaxial electrode, supercapacitor

## Abstract

Carbon layer-coated μm-sized carbon fiber has the potential to be developed as an electrode, as it can be directly used as an electrode without any preparation process in the absence of an insulating binder. In our work, a carbon layer-coated carbon fiber (C@CF) coaxial structure is constructed by in situ conversion of the epoxy resin around the carbon fiber into a carbon layer, in which a sandwich scaffold of cover/CFRP/screen is designed and adopted. The activated SC@CF, i.e., A-SC@CF, can be directly served as the electrode, and has excellent supercapacitor performance: a high specific capacity of 227.1 F g^−1^ at 0.5 A g^−1^, with a capacitance retention of 98.9% after 20,000 cycles for the electrode, and an energy density of 16.68 Wh kg^−1^ at the power density of 1400 W kg^−1^ for its symmetrical supercapacitor (SSC).

## 1. Introduction

Pushed by the demands of both energy collection from sustainable and clean new power sources, including solar, tide and wind, and energy supply for devices including automobiles, tools and electronic devices, energy storage attracts much attention. Due to the fluctuation and intermittence of new energy sources, and power requirements of automobiles and big tools, the supercapacitor, with merits of high power density and an ultralong cycle life, is somewhat superior to the battery [1,2,3,4].

Micrometer-sized carbon fibers can be directly used as electrodes without any preparation process and no insulating binder is involved, which is superior to powdery nano-sized active materials including carbon nanotubes (CNTs) [5], graphene [6], active carbon [7] and carbon aerogels [8] in this aspect. Due to their high conductivity, good chemical stability, light weight and good mechanical property, these μm-sized carbon fibers have been widely used as conductive supports (scaffolds) of the electrode in the supercapacitor, i.e., current collectors [9,10,11,12]. However, they are also insufficient for direct use as electrodes due to two disadvantages: (1) low specific surface area due to their μm-sized diameter and smooth surface, and (2) poor electrochemical activity. It is a challenge to construct μm-sized carbon fiber-based electrodes with high electrochemical performance.

Building nano-sized circular holes [13] and grooves [14] on the carbon fiber surface, as well as decreasing its diameter [15], have proven to be efficient routes to improve its specific surface area. While the mechanics of carbon fiber will undoubtedly be weakened, on the other hand, new carbon fiber is also subject to high costs and high energy consumption. Therefore, coating a carbon layer with high specific surface area and high activity on the surface of the recovered carbon fiber (carbon layer@carbon fiber, C@CF) is an economic and efficient method [16]. However, there have been few reports on this up to now [17,18].

Retired CFRP (carbon fiber-reinforced polymer) has been a rich source of recovered carbon fiber due to its wide application in various fields. Up to now, efficient methods including pyrolysis [19,20], supercritical [21,22], solvothermal [23,24] and microwave [25,26] methods have been used to carefully reclaim clean and undamaged carbon fiber from CFRP, in which bare carbon fiber filaments are obtained; i.e., epoxy resin is entirely (100%) removed. However, this is not an optimized strategy to cover a carbon layer on this reclaimed carbon fiber surface, as epoxy resin is useless and extra efforts are involved. Moreover, energy consumption is not considered.

Herein, a carbon layer-coated carbon fiber (C@CF) coaxial structure is constructed by in situ conversion of the epoxy resin around the carbon fiber into a carbon layer. Under the irradiation and photothermal effect of focused sunlight beams, the epoxy resin becomes molten and is partially removed, and remaining adhesive liquid epoxy resin around the carbon fiber is carbonized and loaded on the carbon fiber surface by the same solar photothermal effect. In particular, when a sandwich structure of ceramic cover/CFRP/stainless screen is designed and used, a coaxial structure of recovered carbon fiber with a uniform carbon layer from epoxy resin is constructed. This C@CF coaxial structure can be directly used as an electrode, and the optimized C@CF coaxial electrode exhibits excellent supercapacitor performance: a high specific capacity of 227.1 F g^−1^ at 0.5 A g^−1^, and a capacitance retention of 98.9% after 20,000 cycles for a single electrode, along with an energy density of 16.68 Wh kg^−1^ at the power density of 1400 W kg^−1^ for its symmetrical supercapacitor (SSC).

## 2. Experimental Section

### 2.1. Reagents and Materials

CFRP plates and the pristine carbon fiber (Shanghai Jinfei Composites Materials Co., Ltd., Shanghai, China) were cut into sizes of 1 cm × 2 cm × 3 mm and 1 cm × 2 cm × 1 mm, respectively. Hydrochloric acid (HCl, 37.5 wt%), potassium hydroxide (≥85.0%), ethanol (≥99.7%) and potassium hydroxide (KOH, AR) were purchased from Scientific Co. Ltd., Shanghai (Shanghai, China). All these reagents were used without further treatment.

### 2.2. Preparation of A-SC@CF Composites

**Figure 1:**
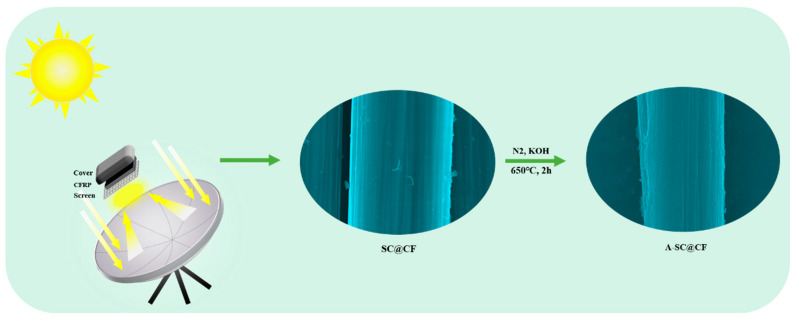
Schematic diagram of the preparation of SC@CFs and A-SC@CFs.

A focused sunlight beam was used to recover SC@RCF from CFRP as the energy source, based on the photothermal effect of a dish-style reflecting and focusing solar generation system with a sunlight attraction sensor, as shown in Figure 1. The system makes the sunlight be focused near the sample stage. The diameter range of the focused solar spot is about 10 cm, and is available from 8:30 am to 5:00 pm.

A dish-style reflecting and focusing solar generation system is used in our work, in which this system has a total area of 1.5 m^2^ to harvest sunlight and a focus area of 0.01 m^2^, and a sunlight concentration ratio of 150. As the sunlight on sunny days has an average power density of 850 W/m^2^ [27], the temperature at the focus can reach the point of up to 600 °C.

Herein, a sandwich structure of CFRP was placed at the solar focus spot, sandwiched with a ceramic cover and a stainless steel screen. The obtained solar-induced carbon layer coated on the carbon fiber samples is named as SC@CF-T-t-m according to the parameters of T, t and m (T: temperature, 400, 450 and 500 °C; t: time, 3, 5 and 7 min; m: mesh number of the stainless steel screen, 10, 20 and 30 meshes). The temperature (T) was controlled by choosing different time intervals from 8:30 am to 5:00 pm. For comparison, SC@CF samples were also tried at 450 °C for 5 min with either a cover or a screen (20 meshes), or neither, and were accordingly termed as SC@CF-S, SC@CF-C and SC@CF-NSC.

After activation at 650 °C in nitrogen for 120 min in KOH at a mass ratio of 1:3, SC@CF samples were converted into A-SC@CF, i.e., A-SC@CF-T-t-m, A-SC@CF-S, A-SC@CF-C and A-SC@CF-NSC, respectively.

### 2.3. Characterization of A-SC@CFs

Scanning electron microscopy (SEM) was performed using a field-emission scanning electron microscope (Hitachi S-4800, Tokyo, Japan). X-ray diffraction (XRD, RIGAKU, Tokyo, Japan, D/MAX 2550 V) was utilized to characterize the crystalline structure of the samples, while Raman spectroscopy (INVIA, Renishaw Instruments, New Mills, UK) was used to determine the chemical changes in the carbon fiber complex material. X-ray photoelectron spectroscopy (XPS, ESCALAB 250 Xi) was performed to determine the chemical states. BET surface area and porosity were determined by N_2_ adsorption/desorption measurements conducted with a Micromeritics ASAP 2020 instrument (Micromeritics Amercian, Inc., Norcross, GA, USA).

### 2.4. Electrochemical Measurements of A-SC@CFs Electrode

As carbon fiber in carbon fiber-reinforced polymer (CFRP) is μm-sized (~7.84 μm, similar to human hair) with an aligned direction and conductive, the reclaimed aligned carbon fiber can be directly used as a flexible electrode once the insulative epoxy resin in CFRP is removed (e.g., SC@CF-NSC) or transformed into carbon (SC@CF-450-5-20). Moreover, activated electrodes, e.g., A-SC@CF-450-5-20, exhibit superior supercapacitor performance. One end of the aligned carbon fiber is immersed in the electrolyte, while the other end is connected with a clip to the electrochemical workstation.

Electrochemical performance, including cyclic voltammetry (CV), galvanostatic current charging−discharging (GCD) and electrochemical impedance spectroscopy (EIS), was evaluated on an electrochemical workstation (CHI660E, Shanghai Chenhua, Shanghai, China) in both two- and three-electrode systems. A group of A-SC@CFs (about 5 mg) was directly used as a working electrode in aqueous solution of 6M KOH, and a platinum foil and Hg/HgO electrode were respectively used as a counter electrode and reference electrode in a standard three-electrode system. Referring to the three-electrode system, the potential window was −1~0 V, while in the two-electrode system, the voltage window was 0~1.4 V. Moreover, in the three-electrode system, scan rates in the cyclic voltammetry (CV) varied from 10 to 100 mV·s^−^^1^, while current densities in the galvanotactic charge and discharge (GCD) varied from 0.5 to 5 A·g^−^^1^ [28,29]. For comparison, in the two-electrode system, scan rates in the CV curves changed from 10 to 200 mV·s^−^^1^, while current densities in GCD curves changed from 0.5 to 10 A·g^−^^1^. The frequency range of electrochemical impedance spectroscopy (EIS) was 10^5^~0.01 Hz with a disturbance of 5 mV.

According to the charge/discharge curves, the specific capacitance of electrodes can be calculated based on the following equation:

(1) $$C_{s}=\frac{I\;\times \;∆t}{\;m\;\times \;∆V}$$

where $C_{s}$ (F g^−1^) is specific capacitance, I (A) is the discharge current, ∆t (s) is the discharge time and ∆V (V) is the discharge voltage range. In the three-electrode system, m (g) represents the mass loading of active material in a single electrode; in a two-electrode cell, m (g) is the mass loading of active materials based on both electrodes.

The energy density (E, Wh kg^−1^) and power density (P, W kg^−1^) are calculated according to the following equations:

(2) $$E\;=\;\frac{C_{s\;}\times \;∆V^{2}}{2\;\times 3.6}$$

(3) $$P=\frac{E}{∆t}\times 3600$$

where $C_{s\;}$ (F g^−1^) is the specific capacitance based on mass loading of active materials in both electrodes. ∆V (V) is the discharge potential range that is exclusive of the IR drop, and ∆t (s) is the discharge time.

## 3. Results and Discussion

### 3.1. Surface Structure of A-SC@CFs

**Figure 2:**
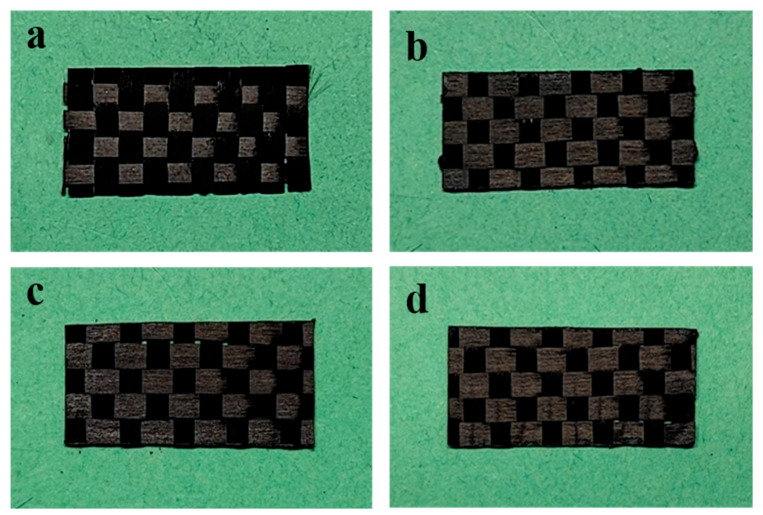
Digital photos of (**a**) SC@CF-NSC, (**b**) SC@CF-S, (**c**) SC@CF-C and (**d**) SC@CF-450-5-20.

Figure 2 presents the digital photos of (a) SC@CF-NSC, (b) SC@CF-S, (c) SC@CF-C and (d) SC@CF-450-5-20. Following the photothermal treatment with concentrated sunshine, there are no discernible alterations in the appearance of these four samples; in other words, their original arrangement has been preserved. In actuality, hard CFRP blocks have been transformed into flexible sheets following the photothermal treatment. Furthermore, the SC@CF-NSC samples exhibit some distinct free carbon fiber filaments, indicating a complete removal of epoxy resin.

**Figure 3:**
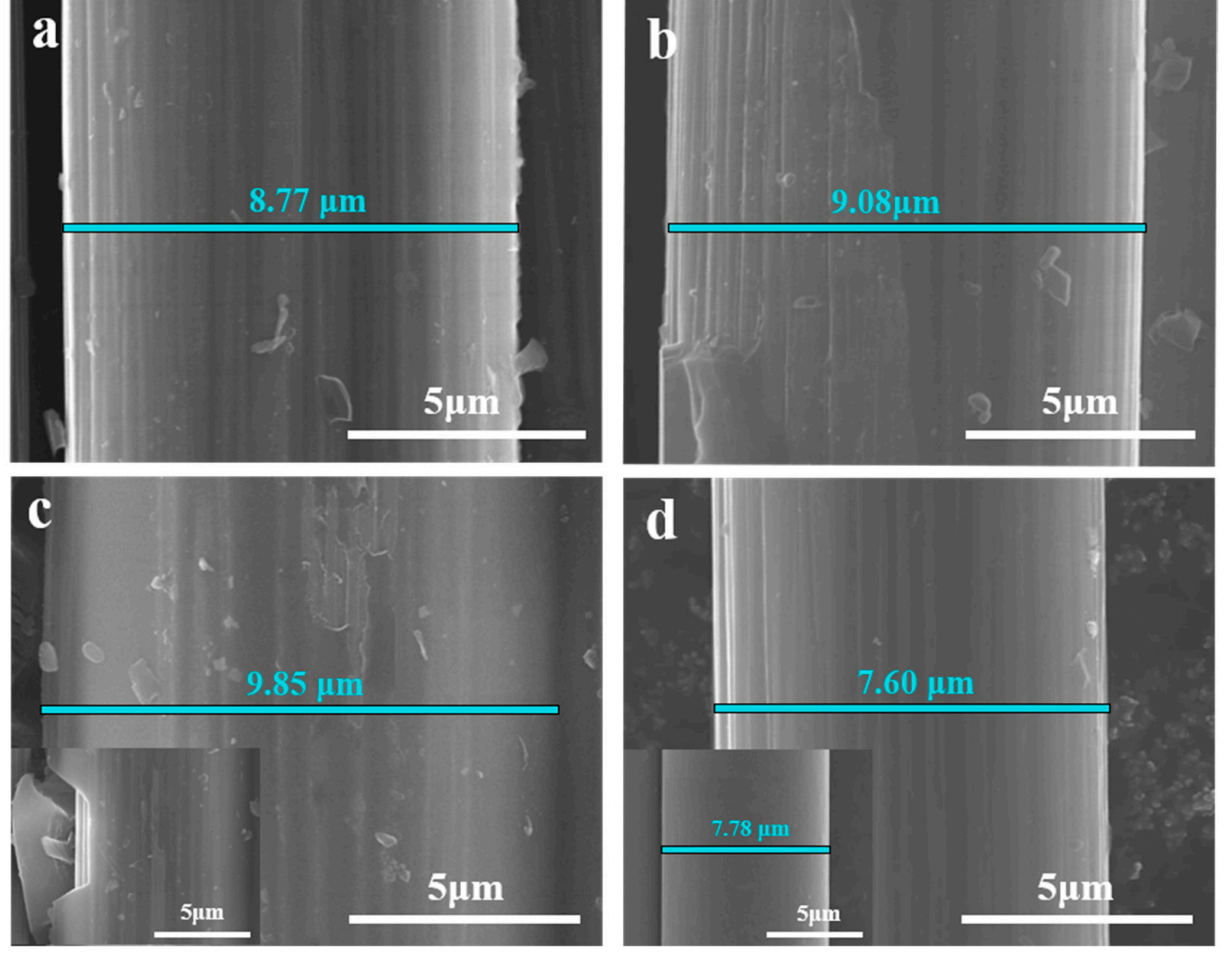
SEM images of (**a**) SC@CF-S, (**b**) SC@CF-C, (**c**) SC@CF-450-5-20 (Inset: enlarged section) and (**d**) SC@CF-NSC (Inset: virgin carbon fiber).

Effects of the cover and screen can be proven by the SEM images. As shown in Figure 3, carbon fiber with distinct grooves along the axis is obtained when neither a ceramic cover nor a stainless steel screen is used (SC@CF-NSC), which is different from the smooth surface of virgin carbon fiber. Moreover, the diameter of SC@CF-NSC (7.60 μm) is slightly smaller than that of virgin carbon fiber (7.78 μm), suggesting the carbon fiber of SC@CF-NSC is partially degraded. However, when a ceramic cover or/and screen is/are involved, the diameters of the samples respectively become 8.77 μm, 9.08 μm and 9.85 μm for SC@CF-S, SC@CF-C and SC@CF-450-5-20, which may be due to the weakening of air intake (AI) caused by the cover or/and the photothermal effect resulting from light intake (LI). Hence, it is reasonable to conjecture that a thin carbon layer is generated on the carbon fiber surface, and the carbon layer of SC@CF-450-5-20 is thicker than those of SC@CF-S and SC@CF-C. Furthermore, this conjecture is confirmed by the split of the carbon layer from the carbon fiber, as shown in a chosen local spot in the Inset in Figure 3c for other SC@CF-450-5-20 samples (Inset of Figure 3c). This solar-induced carbon layer@CF (SC@CF) coaxial structure of SC@CF-S, SC@CF-C and SC@CF-450-5-20 improves the specific surface area and protects the carbon fiber from the electrolyte, and they are thus chosen as the electrode candidates.

**Figure 4:**
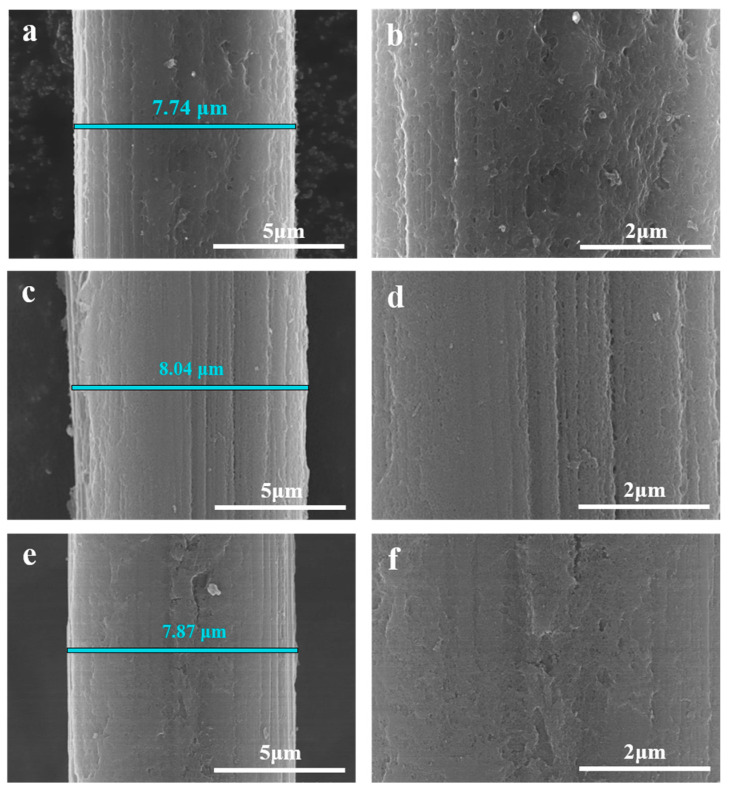
SEM images of (**a**,**b**) A-SC@CF-S, (**c**,**d**) A-SC@CF-450-5-20 and (**e**,**f**) A-SC@CF-C.

As given in Figure 4, the carbon layer is still well kept even after action; e.g., the A-SC@CF coaxial structure is generated for the samples of A-SC@CF-S (Figure 4a,b), A-SC@CF-450-5-20 (Figure 4c,d) and A-SC@CF-C (Figure 4e,f). Their diameters decrease to 7.74, 8.04 and 7.87 μm, which suggests that the carbon layer is partially degraded. Moreover, the diameter of A-SC@CF-450-5-20 (Figure 4c) is bigger than those of A-SC@CF-S (Figure 4a) and A-SC@CF-C (Figure 4e), and the surface of the former is more uniform than those of the latter, suggesting a thicker and more uniform carbon layer for A-SC@CF-450-5-20.

**Figure 5:**
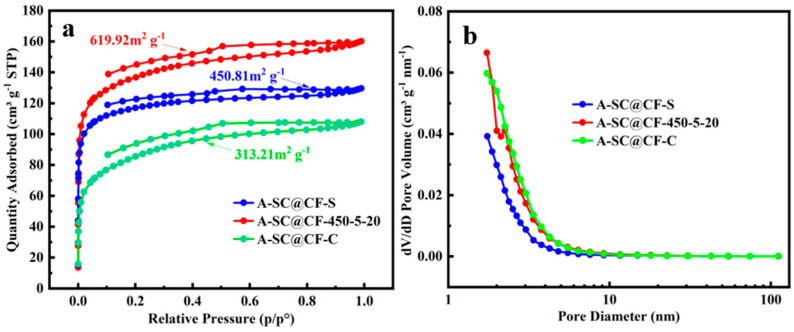
A-SC@CFs samples: (**a**) N_2_ adsorption/desorption isotherms and (**b**) pore size distribution.

Figure 5a,b present the nitrogen adsorption–desorption isotherm and pore size distributions for the three coaxial A-SC@CF samples. It is clearly observed in Figure 5a that the three A-SC@CF samples have similar Type-I adsorption isotherms at low-pressure regions (p/p^0^ < 0.1) and H4-type hysteresis loops at medium pressure (p/p^0^ > 0.4), suggesting the existence of mesopores in the three A-SC@CFs [30]. Furthermore, as listed in Table 1, the specific surface area (619.92 m^2^ g^−^^1^) and pore volume (0.22 cm^3^ g^−^^1^) of A-SC@CF-450-5-20 are higher than those of A-SC@CF-S (450.81 m^2^ g^−^^1^, 0.20 cm^3^ g^−^^1^) and A-SC@CF-C (313.21 m^2^ g^−^^1^, 0.17 cm^3^ g^−^^1^). These indicate that SC@CF-450-5-20 has a superior porosity structure, which is consistent with the SEM images and thus favors its electrochemical performance [31].

### 3.2. Composition of ASC@CFs

**Figure 6:**
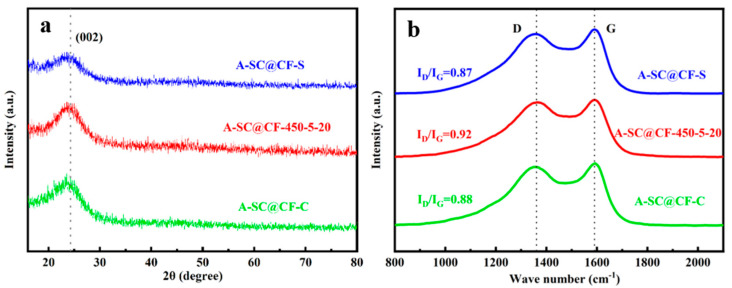
(**a**) XRD patterns and (**b**) Raman spectra of A-SC@CF-S, A-SC@CF-450-5-20 and A-SC@CF-C.

Figure 6a shows XRD patterns of three SC@CF samples, in which the broad peak at 25° attributed to the (002) crystal plane of graphite [32] is clearly observed, indicating an amorphous carbon layer. Moreover, no other characteristic peaks appear in the diffraction pattern, although stainless steel mesh is involved during the sunlight photothermal process, suggesting that no metal composition remains in the SC@CF sample.

As shown in the Raman spectra in Figure 6b, although there exist two bands, the D band and G band, around 1360 and 1585 cm^−^^1^ in all three Raman spectra [33], the I_D_/I_G_ ratio of A-SC@CF-450-5-20 (0.92) is higher than those of A-SC@CF-S (0.87) and A-SC@CF-C (0.88). As the G band is attributed to the stacking of the graphite hexagonal network plane, and the D band is attributed to defects and disordered carbon [34,35], A-SC@CF-450-5-20 has a higher disorder or exfoliation than A-SC@CF-S and A-SC@CF-C [36], which facilitates its electrochemical performance.

**Figure 7:**
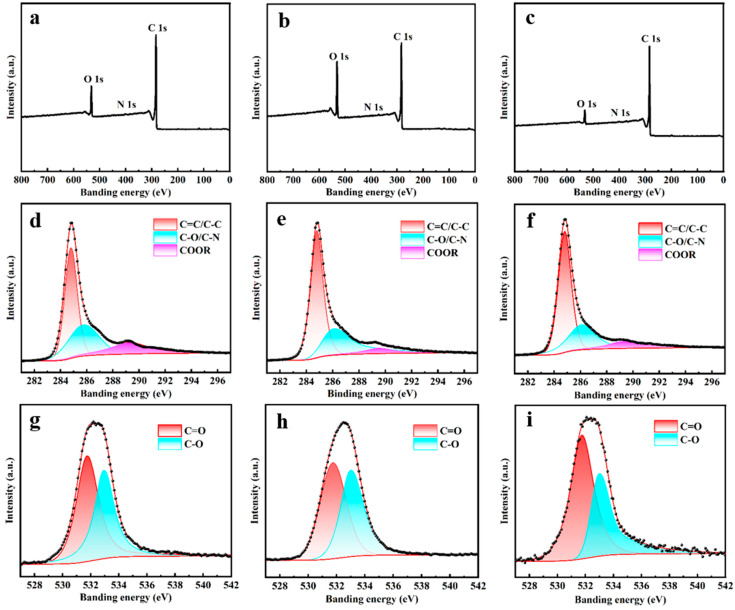
XPS of (**a**,**d**,**g**) A-SC@CF-S, (**b**,**e**,**h**) A-SC@CF-450-5-20 and (**c**,**f**,**i**) A-SC@CF-C for (**a**–**c**) survey spectrum and (**d**–**f**) C 1s and (**g**–**i**) O 1s spectra.

Figure 7 shows XPS of A-SC@CF-450-5-20, and the elemental contents are analyzed and listed in Table 2. C, N and O are found in all three samples, and C and N originated from epoxy resin, while the oxygen is derived from the carbonization of epoxy resin [37] and the oxygen-containing functional groups introduced by the chemical activation process [38]. The high-resolution spectra of C 1s can be fitted to three peaks of 284.8 eV, 285.9 eV and 289.3 eV, which are attributed to C = C/C-C, C-O/C-N and COOR [39], respectively. Moreover, the high-resolution spectra of O 1s are divided into two peaks of 531.7 eV and 532.9 eV, indicating the presence of C = O and C-O groups, which improves the surface hydrophilicity and thus enhances the accessibility of the surface to electrolyte ions [40]. In addition, high oxygen content (23.76%) endows A-SC@CF-450-5-20 with superior activation to A-SC@CF-S and A-SC@CF-C, which will be discussed later.

### 3.3. Electrochemical Performances of A-SC@CF Coaxial Electrode

**Figure 8:**
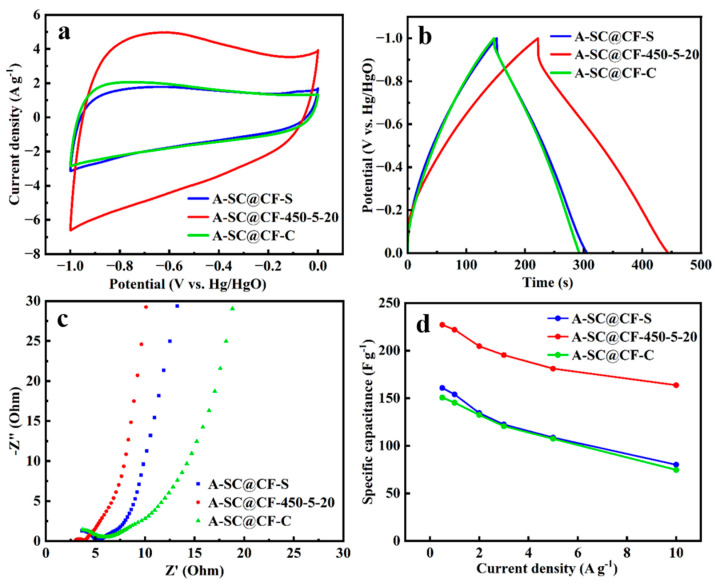
Electrochemical performance of A-SC@CF-S, A-SC@CF-450-5-20 and A-SC@CF-C electrodes in 6 M KOH: (**a**) CV curves at 10 mV s^−^^1^, (**b**) GCD curves at 1 A g^−^^1^, (**c**) Nyquist plots and (**d**) specific capacitances at different current densities.

Figure 8a,b show CV curves and GCD curves of A-SC@CF-S, A-SC@CF-450-5-20 and A-SC@CF-C, respectively. Firstly, rectangle-like CV curves suggest typical electric double layer capacitor (EDLC) behavior [41]. Secondly, the enveloped area in the CV curve of A-SC@CF-450-5-20 is distinctly larger than those of A-SC@CF-S and A-SC@CF-C, indicating the superior specific capacitance of A-SC@CF-450-5-20, which is consistent with its uniform carbon layer in SEM images, high specific surface area in BET and high oxygen content in XPS spectra. The triangle shape of the GCD curves in Figure 8b further confirms the EDLC behavior [42]. According to the GCD curves in Figure 8b and Equation (1), the specific capacitances of A-SC@CF-450-5-20, A-SC@CF-S and A-SC@CF-C are calculated to be 221.9, 152.9 and 145.2 F g^−^^1^, respectively. As shown in EIS in Figure 8c, A-SC@CF-450-5-20 has the smallest X-intercept and a semicircle, suggesting that it has the smallest equivalent series resistance (Rs) and charge transfer internal resistance (Rct). Due to the low Rs and Rct, A-SC@CF-450-5-20 has good rate capability, e.g., 72% retention of the specific capacitance when the current density jumps from 0.5 to 10 A g^−1^, which is higher than A-SC@CF-S (61%) and A-SC@CF-C (59%). For comparison, A-SC@CF-NSC delivers a poor specific capacitance of 84.8 F g^−^^1^, in which neither AI nor LI is controlled. As shown in Figure 3d, compared with virgin carbon fiber (7.78 μm), SC@CF-NSC has a diameter of 7.60 μm, which suggests that all epoxy resin is completely removed. Hence, activated SC@CF-NSC, i.e., A-SC@CF-NSC, has no carbon layer. On the other hand, A-SC@-450-5-20 has a diameter of 8.04 μm; i.e., a thin carbon layer is coated on the surface. These indicate that the electrochemical performance is improved by the carbon layer.

**Figure 9:**
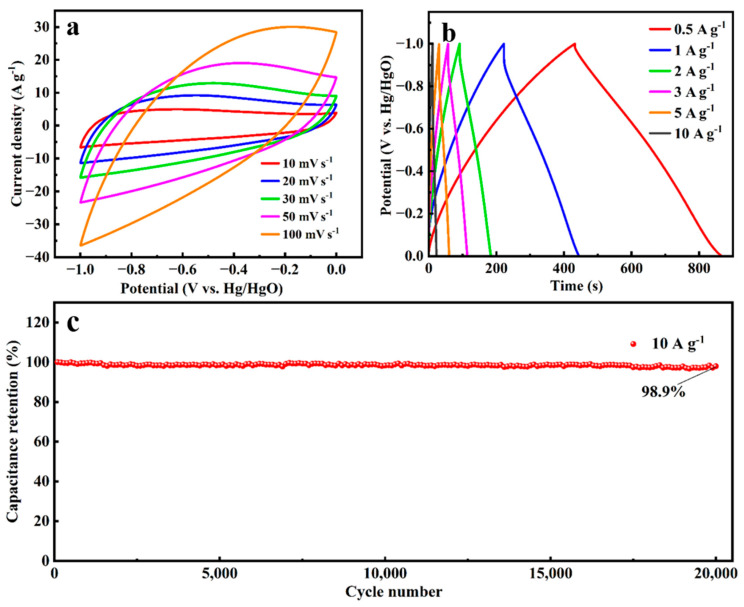
(**a**) CV curves at different scan rates, (**b**) GCD at different current densities and (**c**) cyclability test at 10 A g^−^^1^ for A-SC@CF-450-5-20.

As shown in Figure 9a,b, the ratability of A-SC@CF-450-5-20 is further tested. It is observed that the quasi-rectangular shape of the CV curve is almost maintained even when the scan rate reaches 100 mV s^−^^1^, and the triangle shape of the GCD curve is well kept. In addition, as shown in Figure 9c, 98.9% of the initial specific capacitance is retained even after 20,000 cycles for A-SC@CF-450-5-20; i.e., there is only a 0.000055% decrease per one cycle, suggesting its excellent stability.

The effects of those factors including temperature, duration and screen number on the electrochemical performance of A-SC@CF are evaluated so as to optimize the preparation conditions of the SC@CF samples.

**Figure 10:**
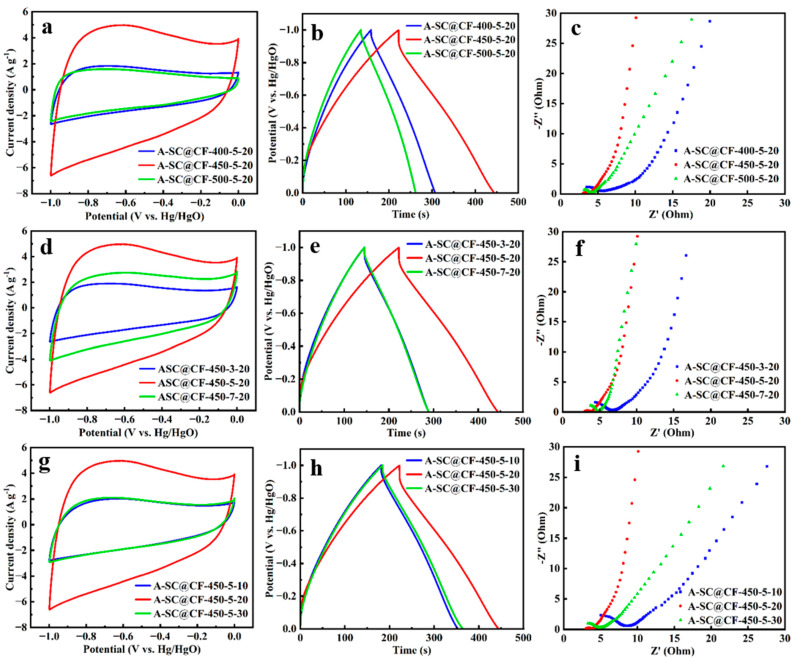
CV curves at 10 mV s^−^^1^, GCD curves at 1 A g^−^^1^ and Nyquist plots of (**a**–**c**) A-SC@CF-T-5-20, (**d**–**f**) A-SC@CF-450-t-20 and (**g**–**i**) A-SC@CF-450-5-m.

Effects of temperature: Besides 450 °C, another two temperatures, 400 °C and 500 °C, are chosen, and their CV curves are compared in Figure 10. Obviously, the CV envelopes of A-SC@CF-400-5-20 and A-SC@CF-500-5-20 are significantly smaller than that of A-SC@CF-450-5-20 (Figure 10a). Based on GCD curves in Figure 10b and Equation (1), the specific capacitances of A-SC@CF-400-5-20 and A-SC@CF-500-5-20 are respectively determined to be 150.6 F g^−^^1^ and 133.1 F g^−^^1^, which are lower than that of A-SC@CF-450-5-20. Moreover, a semicircle in the high-frequency region and vertical line in the low-frequency range are observed, in which the Rs of the intercept with the *X*-axis and the Rct of the diameter of the semicircle of A-SC@CF-450-5-20 are also smaller than those of A-SC@CF-400-5-20 and A-SC@CF-500-5-20 (Figure 10c). As the melting, carbonization (maintenance) and pyrolysis (removal) of epoxy resin are affected by the temperature [43], at 400 °C, although the epoxy resin is melted and coated on the carbon fiber surface, a distorted block rather than uniform carbon layer is formed (Figure S2a). At 500 °C, most epoxy resin is pyrolyzed and the bared carbon fiber is observed (Figure S2b). Hence, 450 °C is the optimized temperature to form a uniform carbon layer on the carbon fiber surface.

Effects of time factors: The time has a similar effect to the temperature on the electrochemical performance. As given in SEM images in Figure S2c,d, when 3 min is used, a distorted block carbon layer is generated on the carbon fiber surface, while bared carbon fiber is observed when 7 min is adopted. As shown in Figure 10d–f, A-SC@CF-450-5-20 (221.9 F g^−^^1^) delivers a superior electrochemical performance (specific capacitance) to other A-SC@CF-450-t-20 samples (i.e., 143.6 F g^−^^1^ for A-SC@CF-450-3-20 and 144.5 F g^−^^1^ for A-SC@CF-450-7-20). Therefore, 5 min is the optimum time to prepare carbon fiber with a uniform carbon layer, i.e., SC@CF-450-5-20. The Rs and Rct of A-SC@CF-450-5-20 are obviously smaller than those of A-SC@CF-450-3-20 and A-SC@CF-450-7-20, which indicates a higher mass transfer velocity. Similarly, the resulting A-SC@CF-450-5-20 exhibits superior electrochemical performances to A-SC@CF-450-3-20 and A-SC@CF-450-7-20.

Effects of mesh number factors: As presented in Figure S2e,f, the screen number also has an influence on the morphology of SC@CF. As screen number affects the LI, intake of sunlight is not enough to produce a photothermal effect to remove all unnecessary epoxy resin when 30 meshes is used, and thus a block carbon layer is produced on the carbon fiber surface. On the other hand, intake of sunlight is too much to remove most epoxy resin, and thus the carbon fiber is only partially coated by the carbon layer. Subsequently, as shown in Figure 10i, the resulting A-SC@CF-450-5-10 (172.4 F g^−^^1^) and A-SC@CF-450-5-30 (180.1 F g^−^^1^) exhibit inferior electrochemical performance to A-SC@CF-450-5-20 (221.9 F g^−^^1^). Accordingly, A-SC@CF-450-5-20 represents the smallest Rs and Rct among the samples (A-SC@CF-450-5-m). Consequently, m = 20 is the best choice.

### 3.4. Electrochemical Performances of SSC of A-SC@CF//A-SC@CF

**Figure 11:**
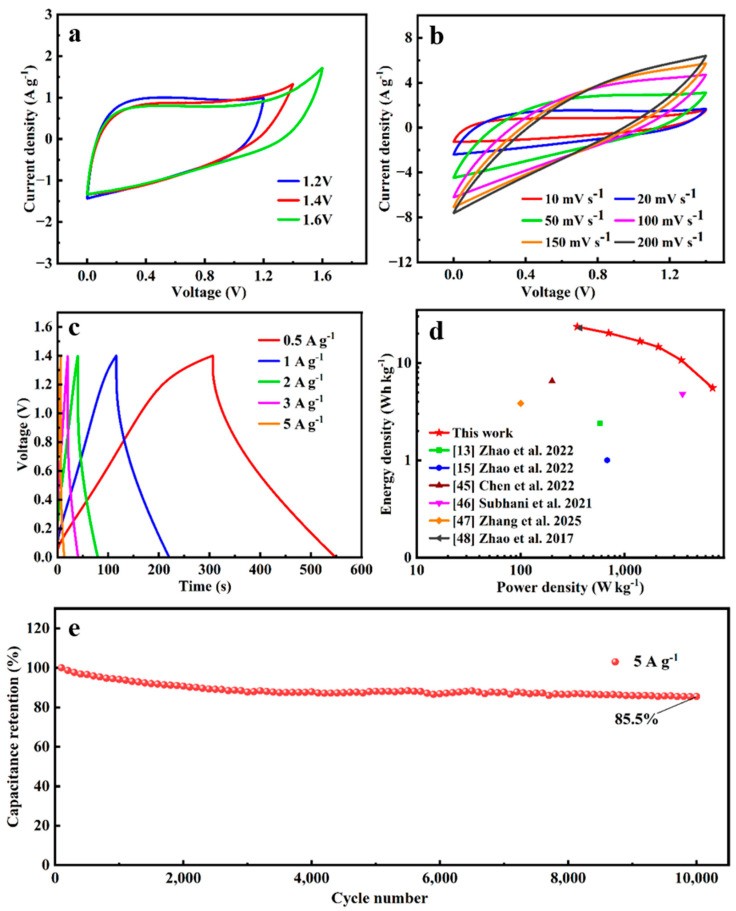
SSC of A-SC@CF-450-5-20//A-SC@CF-450-5-20: CV curves (**a**) within different voltage windows and (**b**) at different scan rates. (**c**) GCD curves with current densities, (**d**) Ragone plots [13,15,45,46,47,48] and (**e**) cyclability.

To further evaluate the practical application of A-SC@CFs, a symmetrical supercapacitor (SSC) was assembled using A-SC@CF-450-5-20 as both the positive and negative electrodes in an electrolyte of 6 M KOH. According to the CV curves in different voltage windows (Figure 11a), 1.4 V is selected as the operating voltage of the SSC [44]. The CV curves almost keep the shape even at 200 mV s^−^^1^, as shown in Figure 10b, suggesting a good ratability. Based on the GCD curves and Equation (1), the specific capacities of SSC at 0.5, 1, 2, 3 and 5 A g^−^^1^ are calculated to be 86.5, 74.6, 61.3, 53.7 and 39.3 F g^−^^1^, respectively. The specific capacity retention of A-SC@CF-450-5-20//A-SC@CF-450-5-20 SSC is over 85% after 10,000 cycles, suggesting good stability. In addition, as given in the Ragone plot (Figure 11d), SSC has an energy density of 23.54 Wh kg^−l^ at a power density of 350 W kg^−1^ and 16.68 Wh kg^−^^1^ at 1400 W kg^−1^, which is a comparable or superior electrochemical performance [13,15,45,46,47,48], as listed in Table 3.

## 4. Conclusions

In sum, assisted by the photothermal effect of a focused sunlight beam, a carbon layer-coated carbon fiber (SC@CF) coaxial structure is constructed by melting, in situ carbonizing the epoxy resin and loading the resulting carbon on the carbon fiber surface. A sandwich structure of ceramic cover/CFRP/stainless screen is designed and used to ensure the uniformity of the carbon layer. According to the adsorption/desorption results, this carbon layer improves the specific surface area. The high I_D_/I_G_ ratio of A-SC@CF-450-5-20 (0.92) in the Raman spectra indicates its high disorder or exfoliation. The high oxygen content (23.76%) in the C = O and C-O groups in XPS improves the surface hydrophilicity and thus enhances the accessibility of the surface to electrolyte ions. The activated SC@CF, i.e., A-SC@CF, can be directly used as the electrode, and has excellent supercapacitor performance: a high specific capacity of 227.1 F g^−^^1^ at 0.5 A g^−^^1^, with a capacitance retention of 98.9% after 20,000 cycles for a single electrode, with an energy density of 16.68 Wh kg^−^^1^ at the power density of 1400 W kg^−^^1^ for its symmetrical supercapacitor (SSC). This work provides a new strategy to construct μm-sized carbon fiber-based supercapacitor electrodes with good electrochemical performance.

## Figures and Tables

**Table 1:** The specific surface area and volume of A-SC@CF-S, A-SC@CF-450-5-20 and A-SC@CF-C.

Sample	S_BET_ (m^2^ g^−1^)	Pore Volume (cm^3^ g^−1^)
A-SC@CF-S	450.81	0.20
A-SC@CF-450-5-20	619.92	0.22
A-SC@CF-C	313.21	0.17

**Table 2:** Element analysis of the SC@CF samples (wt%).

Sample	C 1s	O 1s	N 1s
A-SC@CF-S	85.55	13.96	0.49
A-SC@CF-450-5-20	75.83	23.76	0.42
A-SC@CF-C	88.84	10.24	0.92

**Table 3:** Comparison of A_SC@CF and several reported electrode performances.

Samples	Energy Density (Wh kg^−1^)	Ref.
A-SC@CF-450-5-20	23.54	This work
FHRCF-7-155//FHRCF-7-155	3.84	[13]
AVCF//AVCF	6.5	[15]
ACFP-15//ACFP-15	1	[45]
GACF//GACF	2.4	[46]
N-PC/HWCF-800//N-PC/HWCF-800	4.8	[47]
MRCF//AC	22.9	[48]

## Data Availability

The original contributions presented in this study are included in the article; further inquiries can be directed to the corresponding author.

## Supplementary Materials

The following supporting information can be downloaded at https://www.mdpi.com/article/10.3390/molecules30153093/s1, Figure S1. Digital photos of (a) SC@CF-400-5-20, (b)SC@CF-500-5-20, (c) SC@CF-450-3-20, (d) SC@CF-450-7-20, (e)SC@CF-450-5-30 and (f) SC@CF-450-5-10. Figure S2. SEM images of (a) ASC@CF-400-5-20, (b) ASC@CF-500-5-20, (c) ASC@CF-450-3-20, (d) ASC@CF-450-7-20, (e) ASC@CF-450-5-30 and (f) ASC@CF-450-5-10. Table S1. Performance comparison between A-SC@CF-450-5-20 and several carbon-based electrodes. References [15,41,42,49] are cited in the Supplementary Materials.
