# Supplementary material for: Focused Solar-Induced Construction of Activated Solar Carbon@Carbon Fiber Coaxial Electrode from Waste Carbon Fiber-Reinforced Polymer and Its Supercapacitor Performance
Source: Molecules. 2025 Jul 24;30(15):3093. doi: 10.3390/molecules30153093 (PMC12348260; doi:10.3390/molecules30153093)
Supplement: Supplementary file 1 [file molecules-30-03093-s001.zip › molecules-3747903-supplementary.pdf]

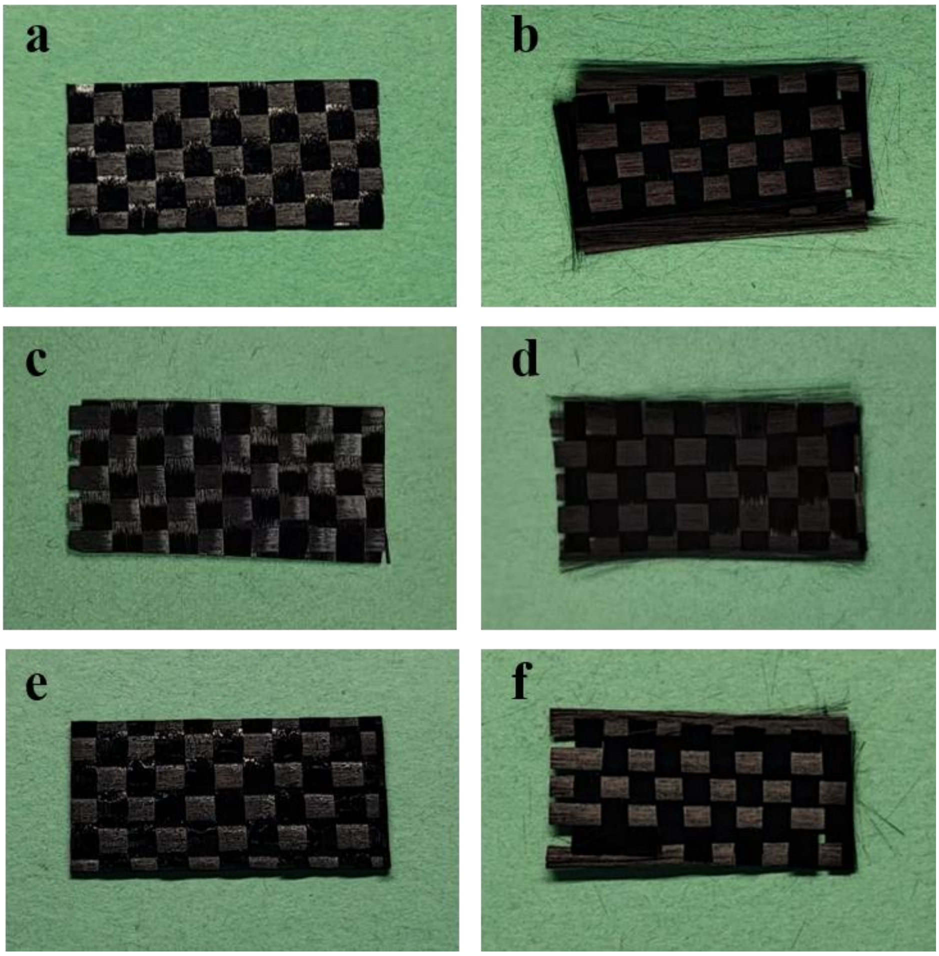

Figure. S1. Digital photos of (a) SC@CF-400-5-20, (b)SC@CF-500-5-20, (c) SC@CF-450-3-20, (d) SC@CF-450-7-20, (e)SC@CF-450-5-30 and (f) SC@CF-450-5-10.

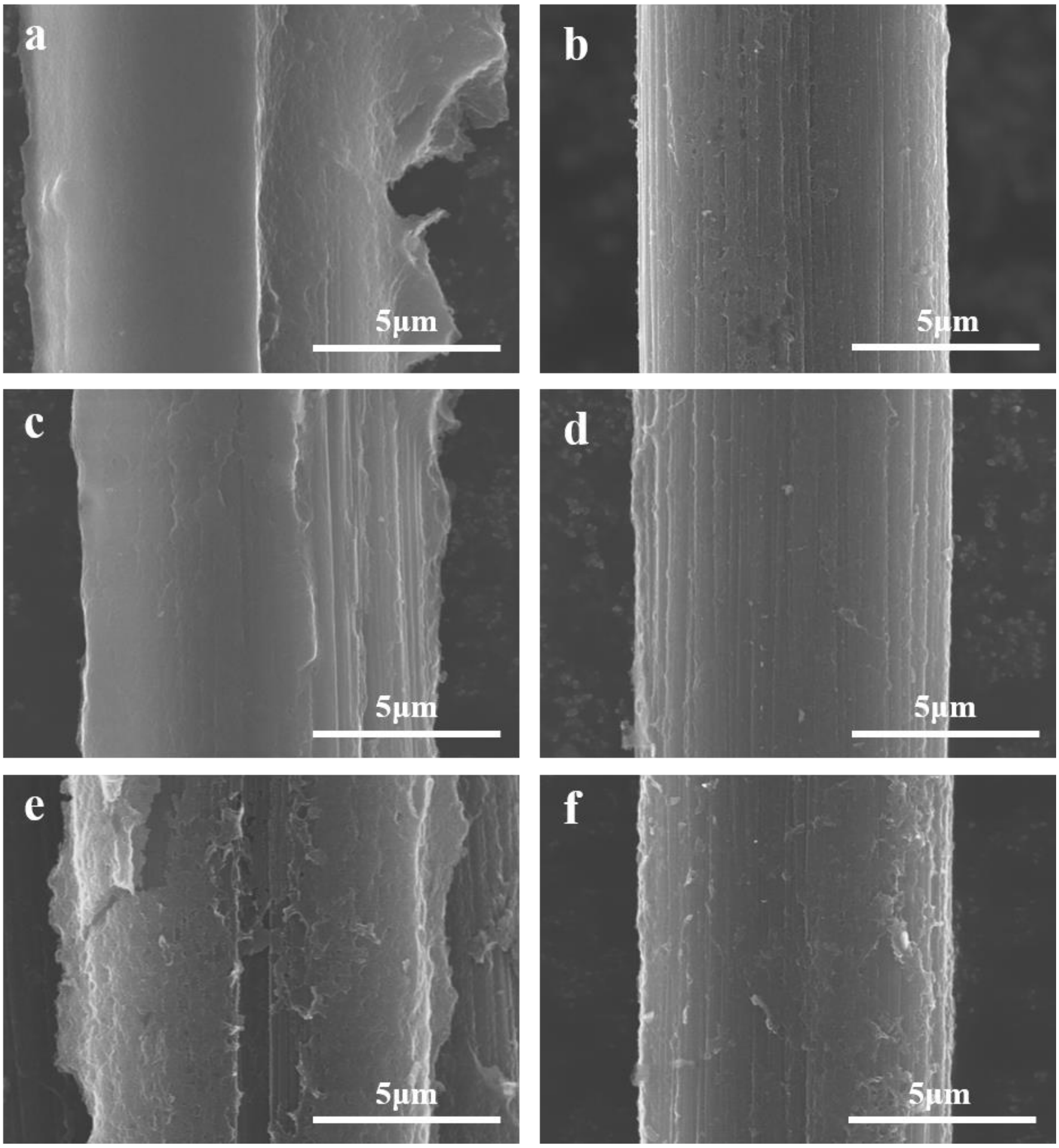

Figure. S2. SEM images of (a) ASC@CF-400-5-20, (b) ASC@CF-500-5-20, (c) ASC@CF-450-3-20, (d) ASC@CF-450-7-20, (e) ASC@CF-450-5-30 and (f) ASC@CF-450-5-10.

Table S1 Performance comparison between A-SC@CF-450-5-20 and several carbon-based electrodes

Material	Specific capacitance ($F\ g^{-1}$)	Cyclic stability	Energy density ($Wh\ kg^{-1}$)	Ref.
CC-F1	42.2	92%(1000 cycles)	4.64	[1]
AVCF	118.97	90.8%(10000 cycles)	6.5	[2]
CSC-800	402.8	96.6%(20000 cycles)	26.6	[3]
PW-700	520	99%(40000 cycles)	21.5	[4]
A-SC@CF-450-5-20	221.9	98.9%(20000 cycles)	23.54	This work

Reference

[1] Pang, S.; Lin, L.; Shen, Y.; Chen, S.; Chen, W.; Tan, N.; Ahmad, A.; Al-Kahtani, A. A.; Tighezza, A. M. Surface Activated Commercial Carbon Cloth as Superior Electrodes for Symmetric Supercapacitors. Materials Letters 2022, 315, 131985. https://doi.org/10.1016/j.matlet.2022.131985.

[2] Zhao C, Zheng J, Wang Y, et al. Vaporized hydrothermal functionalization of carbon fiber and its superior supercapacitor performance [J]. Energy & Fuels, 2022, 36(7): 4052-4064.

[3] Wan, L.; Li, X.; Li, N.; Xie, M.; Du, C.; Zhang, Y.; Chen, J. Multi-Heteroatom-Doped Hierarchical Porous Carbon Derived from Chestnut Shell with Superior Performance in Supercapacitors. J. Alloys Compd. 2019, 790, 760−771.

[4] Sandhiya, M.; Nadira, M. P.; Sathish, M. Fabrication of Flexible Supercapacitor Using N-Doped Porous Activated Carbon Derived from Poultry Waste. Energy Fuels 2021, 35 (18), 15094–15100. https://doi.org/10.1021/acs.energyfuels.1c01713.
